# Influence of patients’ disease knowledge and beliefs about medicines on medication adherence: findings from a cross-sectional survey among patients with type 2 diabetes mellitus in Palestine

**DOI:** 10.1186/1471-2458-14-94

**Published:** 2014-01-30

**Authors:** Waleed M Sweileh, Sa’ed H Zyoud, Rawan J Abu Nab’a, Mohammed I Deleq, Mohammed I Enaia, Sana’a M Nassar, Samah W Al-Jabi

**Affiliations:** 1 Department of Pharmacology/ Toxicology, College of medicine and health sciences, An-Najah National University, Nablus, Palestine; 2 Department of clinical pharmacy and Pharmacotherapy, College of medicine and health sciences, An-Najah National University, Nablus, Palestine; 3 Pharm. D Program, College of medicine and health sciences, An-Najah National University, Nablus, Palestine

**Keywords:** Chronic illness, Beliefs about medicines, Adherence, Palestine

## Abstract

**Background:**

Diabetes mellitus (DM) is a common serious health problem. Medication adherence is a key determinant of therapeutic success in patients with diabetes mellitus. The purpose of this study was to assess medication adherence and its potential association with beliefs and diabetes – related knowledge in patients with type II DM.

**Methods:**

This study was carried out at Al-Makhfia governmental diabetes primary healthcare clinic in Nablus, Palestine. Main outcome of interest in the study was medication adherence. The Beliefs about Medicines Questionnaire (BMQ) was used to assess beliefs. Morisky Medication Adherence Scale (MMSA-8^©^) was used to assess medication adherence. The Michigan diabetes knowledge test (MDKT) was used to assess diabetes – related knowledge. Univariate and multivariate analysis were carried out using Statistical Package for Social Sciences (SPSS 20).

**Results:**

Four hundred and five patients were interviewed. The mean ± SD age of the participants was 58.3 ± 10.4 (range = 28 – 90) years. More than half (53.3%) of the participants were females. Approximately 42.7% of the study sample were considered non-adherent (MMAS-8^©^ score of < 6). Multivariate analysis showed that the following variables were significantly associated with non-adherence: disease-related knowledge, beliefs about necessity of anti-diabetic medications, concerns about adverse consequences of anti-diabetic medications and beliefs that medicines in general are essentially harmful. Diabetic patients with high knowledge score and those with strong beliefs in the necessity of their anti-diabetic medications were less likely to be non-adherent ([O.R = 0.87, 95% CI of 0.78 – 0.97] and [O.R = 0.93, 95% of 0.88 – 0.99] respectively). However, diabetic patients with high concerns about adverse consequences of anti-diabetic medications and those with high belief that all medicines are harmful were more likely to be non-adherent ([O.R = 1.09; 95% C.I of 1.04 – 1.16] and [O.R = 1.09, 95% C.I of 1.02 – 1.16] respectively).

**Conclusions:**

Beliefs and knowledge are important factors in understanding variations in medication adherence among diabetic patients. The BMQ can be used as a tool to identify people at higher risk of non-adherence. Improving knowledge of patients about their illness might positively influence their medication adherence.

## Background

Diabetes mellitus (DM) is a common health problem that has serious medical and economic consequences. The world prevalence of diabetes among adults (aged 20–79 years) was estimated to be 6.4% in 2010 and will increase to 7.7% by 2030 [1]. Between 2010 and 2030, there will be a 69% increase in numbers of adults with diabetes in developing countries and a 20% increase in developed countries. It is striking that Arab world (North Africa, Middle East, and Gulf area) will have the second highest increase in percentage of people with DM in 2030 compared to other parts of the world [1]. No reliable data about treatment outcomes, complications, and economic effects of diabetes mellitus are available from Middle East in general and from Palestine in particular [2].

Medication adherence has important therapeutic and economic consequences [3,4]. Medication adherence is believed to be influenced by factors beyond the traditional demographic and clinical factors [5,6]. For example, the extended Self-Regulatory Model, which includes both illness and treatment beliefs, was successful in explaining variations in medication adherence among patients with certain chronic diseases [7]. Diabetes-related knowledge have also been reported to influence both medication adherence and glycemic control [8]. This suggests that there is a complex model of demographic, clinical, knowledge and behavioral factors that affect medication adherence.

Several studies were carried out in Palestine and Arab world about medication adherence in general and among diabetic patients in particular [9,10]. Unfortunately, none of these studies investigated the influence of factors such as disease-related knowledge or behavioral aspects on medication adherence. Such factors are important given the cultural differences between Arabs and Europeans or Asians where most studies about influence of behavioral and knowledge aspects on medication adherence were carried out [6,11,12]. Beliefs about medicines and extent of disease-related knowledge are different among different cultures [13]. Therefore, the objective of this study was to assess anti-diabetic medication adherence and its potential association with beliefs and diabetes–related knowledge among patients with type II DM attending a primary healthcare clinic in Palestine.

## Methods

### Study design

This was a cross-sectional study for the purpose of evaluating the association between beliefs about medicines, diabetes-related knowledge, demographic and clinical factors with medication adherence among Palestinians with type 2 diabetes mellitus. The approach we followed in this study was similar to that used by other scholars who investigated the relationship between medication adherence and other factors [14-17]. The research approach was based on using medication adherence as an outcome (adherence versus non-adherence) while using demographic, clinical, psychological factors as independent variables. The tools used in this study have been previously used by other investigators [14-21].

### Study setting

Nablus is the largest city in north West-Bank of Palestine. Residents of Nablus city are predominantly Arabs. The study was carried out at Al-Makhfia governmental diabetes primary healthcare clinic in Nablus city. Al-Makhfia center is the main governmental center that provides care for diabetic patients with governmental insurance in Nablus city. During the study period, the investigators made daily visits to the diabetic clinic to recruit and interview potential participants.

### Sample population

This study included a convenience sample of adult population. Participants were recruited from Al-Makhfia diabetic clinics while waiting to be seen by their health care providers. The inclusion criteria for this study were: 1) patients who reported having type 2 diabetes; 2) availability of a medical file at the diabetic clinic; 3) a history of at least one year of diabetes mellitus; 4) currently being under medical care for diabetes; and finally 5) willingness to participate in this study. The main exclusion criterion was physical and/or mental conditions that could interfere with the participant’s ability to understand and/or answer questions in any of the used scales.

### Sample size

A previous study indicated that medication non-adherence among Palestinian diabetic patients is in the range of 20 – 50% [9]. Therefore, the sample size was estimated based on the following assumptions: a descriptive study with dichotomous outcome (adherence versus non-adherence), rate of medication non-adherence to be as low as 20%, confidence interval width of 10% and confidence limit to be 95%. Therefore, an estimated sample of 385 diabetic patients is needed for this study [22]. In order to minimize erroneous results and increase the study reliability, the investigators recruited a total of 405 diabetic patients during the study period between July 2012 and October 2012.

### Ethical approval

This study was approved by the Palestinian Ministry of Health and Institutional Review Board (IRB) at An-Najah National University. The interviewer explained the purpose of the study to each participant and a verbal consent was obtained from the participants prior to the commencement of the study. The participants were also informed that their participation was voluntary and that they could withdraw from the interview at any time without consequences. The participants were assured that their responses would be treated in confidence and they were assured anonymity through the use of strict coding measures. All information was kept confidential. The study was carried out in full compliance with the guidelines of good clinical practice of the world assembly declaration of Helsinki and was approved by the university ethical committee.

### Recruitment procedure

A brief screening was conducted by investigators to identify potential participants in the following manner: every person in the waiting area of the diabetic clinic was asked if he/she is willing to talk to the investigator. If the person agreed to talk to the investigator for possible participation, then an informed consent was read and obtained by the investigators. Once the verbal consent was obtained, verification of inclusion and exclusion criteria took place. The questionnaires required for the study were presented and explained during this interview. All participants completed the questionnaires in a private area in the clinic.

The forms and questionnaires used in this study were: 1) demographic and clinical information about the participant, 2) Morisky Medication Adherence Scale (MMAS-8^©^) to determine level of medication adherence, 3) Beliefs about Medicines (BMQ) to assess beliefs of participants about their medications; and 4) Michigan Knowledge scale to gain information about diabetes-related knowledge among participants.

### Instruments

#### ***Medication adherence***

Adherence to anti-diabetic medications was measured using Morisky Medication Adherence Scale (MMAS-8^©^) [23]. Approval to use and translate the (MMAS-8^©^) into Arabic language was obtained from the developer. The translation was carried out according to standard forward and backward method. The Arabic- translated version of (MMAS-8^©^) was used in previous publication [9]. The (MMAS-8^©^) consists of eight questions designed to measure medication adherence. The first 7 ones are Yes/No questions while the last question is answered on a 5-point Likert scale. One point is given for each sentence based on the answer. In the first 7 questions, one point is given for each “NO” answer except for question number 5 where one point is given for the “YES” answer. For item number 8, one point is given for “never/rarely” item and zero point is given for “all the time” item. The total MMAS-8^©^ score is the summation of the scores for the 8 questions. The total score obtained ranges from 0–8. In this study, patients with a total score of MMAS-8^©^ < 6 were considered non-adherent.

#### ***Beliefs about medicines***

Beliefs about Medicines was assessed using Beliefs about Medicines Questionnaire (BMQ); [11]. Approval to use and translate BMQ was obtained from the developer. Translation of BMQ was also carried out according to the standard forward and backward method. BMQ consists of two sections, general and specific. The Specific section assesses patients’ beliefs about medications prescribed for a particular illness and consists of two scales that assess personal beliefs about the *necessity* and *concerns* of prescribed medication for controlling their illness (5 statements for each scale). The General section deals with more general beliefs about medicines and consists of two scales, the *General-overuse* scale which addresses views about the way in which medicines are used by physicians (4 statements), and the *General-harm* scale which assesses beliefs about the degree to which patients perceive medicines as essentially harmful. Each statement has five potential answers (strongly disagree, disagree, uncertain, agree, and strongly agree). The answers are scored from 1 (strongly disagree) to 5 (strongly agree). Points of each scale are summed to give a scale score. Higher scores indicate stronger beliefs in the concepts of the scale.

There are five statements in Specific-necessity and Specific-concerns scales and therefore the total sum of possible scores in these scales would range from 5 to 25. Higher specific-necessity scores represent stronger perceptions of personal need for the medication to maintain health now and in the future. Higher specific-concerns scores represent stronger concerns about the potential negative effects of the medication. There are four statements in the General-overuse and the General-harm scales and therefore the total sum of possible scores in these scales would range from 4 to 20. Higher scores on the General-harm scale represent more negative views about medicines as a whole and a tendency to see medicines as fundamentally harmful and addictive poisons. Higher scores on the General-overuse indicate more negative views about the way in which medicines are prescribed and beliefs that they are overused by physicians.

#### ***Diabetes knowledge test***

The brief diabetes knowledge test developed by the Michigan Diabetes Research and Training Center (MDRTC) known as Michigan diabetes knowledge test (MDKT) [24] was translated into Arabic and used to assess the general knowledge of the participants. Approval to use and translate MDKT was obtained from the developer. The general MDKT consists of 14 multiple choice questions with one correct choice for each question. The Knowledge score is determined by giving one point for each correct answer and zero for the wrong answer or no response. Those who get the highest scores are the most knowledgeable about diabetes. The total knowledge scores ranges from 0 to 14 with higher scores indicating higher level of diabetes general knowledge. The diabetes knowledge test is an effective, efficient, and inexpensive way to obtain a general assessment of a patient's knowledge about diabetes and its care. Due to the fact that the test is short and its reading level is that of the 6^th^ grade level, the diabetes knowledge test can usually be self-administered. Moreover, review of correct and incorrect items also can be used to provide feedback to patients and creates opportunities for teachable moments [24].

#### ***Demographic and health information***

This section of the questionnaire was designed to obtain information about demographic data and health history of the participants. This section included information about age, gender, years of education, income, medical history, and current anti-diabetic medications. The medical history question was presented to the participant as a list of nine illnesses with dichotomous (yes/no) response. All the participants were asked to report all the medications that they use on chronic basis. The data reported by the participants regarding their anti-diabetic medications was validated through checking the computerized system at the MOH which contained up-to-date information about the patients and their medications.

### Data management and statistical analysis

During the pre-analysis phase, the data were coded to maintain confidentiality for all participants. Summative score of the instruments was entered as a continuous measure. Data were entered and analyzed using SPSS (Statistical Package for the Social Sciences). Descriptive statistics was carried out for all variables and expressed as mean ± SD for continuous variables with normal distribution and as median (inter-quartile range: Q1-Q3) for non-normally distributed variables. Kolomogrov-Smirnov test was used to test normality of the variables. Factors associated with non-adherence (MMAS-8 score < 6) were analyzed with binary logistic regression followed by multiple logistic regression analysis. The dependent variable was non-adherence (coded as 1). Multiple logistic regression analysis was carried out using variables that showed significance in univariate analysis. A p value of < 0.05 was considered statistically significant. The relationship between non-adherence and any particular variable of interest was evaluated by calculating an odds ratio (O.R) at 95% confidence interval (C.I).

## Results

### General characteristics of the study sample

During the study period, a total of 405 consecutive patients were recruited. The mean ± SD age of the participants was 58.3 ± 10.4 (range = 28 – 90) years. More than half of the participants were females (216; 53.3%). Patients reported an average of 2.1 ± 1.6 additional illnesses (median = 4; Q1 - Q3: 3 – 6). Hypertension (187; 46.2%), and dyslipidemia (149; 36.8%) were the most frequently reported additional illnesses in the study sample. Patients reported an average of 1.8 ± 0.7 (median = 2; Q1 – Q3: 1 – 2) anti-diabetic medications and 4.3 ± 2.1 (median = 5; Q1-Q3: 4 – 7) different medications taken on daily basis. One hundred and sixty eight patients (41.5%) reported taking ≥ 5 medications on a regular basis. The mean duration of DM reported by the patients was 7 ±6 (median = 8.5; Q1-Q3: 4 – 12 years). More than half of the participants had ≤ high school education (334; 82.5%). Demographic and clinical characteristics of participants are presented in Table 1.

**Table 1:** Univariate analysis of factors associated with non-adherence

**Variable**	**Total N = 405**	**Non-adherent N = 173**	**Adherent N = 232**	**Odds ratio with 95% CI**	**P-value**
**Age**	58.3 ± 10.4	59.0 ± 10.7	57.7 ± 10.2	1.0 (0.99 – 1.0)	0.21
**Gender**					0.58
Male	189 (46.7%)	78 (45.1%)	111 (47.8%)	Reference	
Female	216 (53.3%)	95 (54.9%)	121 (52.2%)	1.1 (0.8 – 1.7)	
**Education**					0.54
Illitrate	49 (12.1%)	23 (13.3%)	26 (11.2%)	Reference	
Primary	168 (41.5%)	73 (42.2%)	95 (40.9%)	0.9 (0.5 – 1.6)	
Secondary	117 (28.5%)	52 (30.1%)	65 (28.0%)	0.9 (0.5 – 1.8)	
College	71 (17.5%)	25 (14.5%)	46 (19.8%)	0.6 (0.3 – 1.3)	
**Marital Status**					0.021
Single	105 (25.9%)	55 (31.8%)	50 (47.6%)	Reference	
Married	300 (74.1%)	118 (68.2%)	182 (60.7%)	0.6 (0.4 – 0.9)	
**Disease knowledge score**	8 (7 – 10)	8 (6 – 9)	9 (7 – 10)	0.8 (0.7 – 0.9)	< 0.001
**Duration of Diabetes Mellitus**	8.5 ± 6.0	8.5 ± 5.8	8.5 ± 6.2	1.0 (0.96 – 1.0)	0.96
**Presence of chronic diseases**					0.02
Yes	284 (70.1%)	132 (46.5%)	152 (53.5%)	1.7 (1.1 – 2.6)	
No	121 (29.9%)	41 (33.9%)	80 (66.1%)	Reference	
**Anti-diabetic therapy**					0.34
Monotherapy	144 (35.6%)	57 (32.9%)	87 (37.5%)	Reference	
Combination	261 (64.4%)	116 (67.1%)	145 (62.5%)	1.2 (0.8 – 1.8)	
**Insulin Use**					0.32
Yes	132 (32.6%)	61 (35.3%)	71 (30.6%)	Reference	
No	273 (67.4%)	112 (64.7%)	161 (69.4%)	0.8 (0.5 – 1.2)	
**Number of medications**	4.3 ± 2.1	4.5 ± 2.1	4.1 ± 2.1	1.1 (1.0 – 1.2)	0.042
Specific -necessity score	18.5 ± 4.0	17.9 ± 4.1	19.0 ± 3.8	0.94 (0.9 – 1.0)	0.009
Specific- concern score	14.0 ± 4.3	15.2 ± 3.9	13.1 ± 4.3	1.1 (1.1 – 1.2)	< 0.001
General – overuse score	12.0 ± 3.3	12.0 ± 3.3	12.0 ± 3.3	1.0 (0.94 – 1.1)	0.95
General – harm score	10.5 ± 3.7	11.5 ± 3.7	10.0 ± 3.7	1.1 (1.1 – 1.2)	< 0.001

*Abbreviations*: *C.I* confidence interval.

### Reported adherence and beliefs

The internal consistency of the MMAS-8^©^ was satisfactory (alpha = 0.7). Two hundred and thirty two (57.3%) patients were considered adherent (MMAS-8^©^ adherence score ≥ 6) while 173 (42.7%) were non-adherent (MMAS-8^©^ adherence score < 6). More than one third (38%) of the participants reported that sometimes they forgot to take their anti-diabetic medications; 24.0% reported that they did not take their medications on at least one occasion in the two weeks before the interview; 17.0% reported that they discontinued taking their medications without telling their doctor when they felt worse upon taking their medications; 33.1% reported that they sometimes forgot to take their medications with them when they traveled or left home; 91.4.0% reported taking all their medications on the day before the interview; 17.0% reported that they stopped taking their medicines when they felt like their diabetic symptoms were under control; 34.6% reported feeling hassled by their treatment plans; and finally 73.9% reported that they had difficulty remembering to take all their medicines at least once in a while (Table 2). Internal consistency for the BMQ four subscales was acceptable and showed values between alpha = 0.63 – 0.82. Scores for Specific-necessity subscale were significantly higher than that for Specific-concern scores [median (interquartile): 20 (16 – 21) versus 14 (10–17); p < 0.001]. The medians (interquartile) for General-overuse and General-harm subscale were 12 (10 – 14) and 10 (8 – 13) respectively. Participants endorsed the belief that their medications are necessary for their current health but they were concerned about becoming too much dependent on their medications. Fifty one percent of the participants who endorsed belief that medications are harmful were non-adherent. Finally, analysis of MDKT scores showed that the majority of the participants (327; 80.7%) scored ≥ 7 out of a total score of 14. The mean ± SD of the MDKT scores was 8.2 ± 2 and median (Q1 – Q3) of 8 (7 – 10).

**Table 2:** **Self-reported medication adherence behavior of study participants as determined by the Morisky 8-Item Medication Adherence Scale (MMAS-8**^**©**^**)**

**Item**	**Number (%) of patients who answered yes**
Do you sometimes forget to take your [health concern] pills?	154 (38.0%)
People sometimes miss taking their medications for reasons other than forgetting. Thinking over the past two weeks, were there any days when you did not take your [health concern] medicine?	97 (24.0%)
Have you ever cut back or stopped taking your medication without telling your doctor, because you felt worse when you took it?	69 (17.0%)
When you travel or leave home, do you sometimes forget to bring along your [health concern] medication?	134 (33.1%)
Did you take your [health concern] medicine yesterday?	370 (91.4%)
When you feel like your [health concern] is under control, do you sometimes stop taking your medicine?	69 (17.0%)
Taking medication everyday is a real inconvenience for some people. Do you ever feel hassled about sticking to your [health concern] treatment plan?	140 (34.6%)
How often do you have difficulty remembering to take all of your medicine?	
Never/rarely	125 (30.9%)
Once in a while	174 (43%)
Sometimes	86 (21.2%)
Usually	14 (3.5%)
All the time	6 (1.5%)

### Factors affecting non-adherence

Univariate analysis (Table 1) showed that there was no significant difference between adherers and non-adherers in age, duration of illness, gender, education, anti-diabetic regimen and use of insulin. However, there was a significant association between non-adherence and marital status, presence of other chronic illnesses, diabetes-related knowledge, total number of chronic medications, specific-necessity, specific-concern and general-harm scales. Married participants and those with high diabetes-related knowledge score were less likely to be non-adherent ([O.R = 0.6; 95% C.I of 0.4 – 0.9] and [O.R = 0.8; 95% C.I of 0.7 - 0.9] respectively). Adherers have significantly higher specific-necessity, lower specific-concern, and lower general-harm scores compared to non-adherers. Multivariate analysis (Table 3) showed that the following variables were significantly associated with non-adherence: disease-related knowledge, beliefs about necessity of anti-diabetic medications, concerns about adverse consequences of the chronic use of anti-diabetic medications and beliefs that medicines in general are essentially harmful. Diabetic patients with high knowledge score and those with strong beliefs in the necessity of anti-diabetic medications were less likely to be non-adherent ([O.R = 0.87, 95% CI of 0.78 – 0.97] and [O.R = 0.93, 95% of 0.88 – 0.99] respectively). However, diabetic patients with high concerns about adverse consequences of anti-diabetic medications and those with high belief that all medicines are essentially harmful were more likely to be non-adherent ([O.R = 1.09; 95% C.I of 1.04 – 1.16] and [O.R = 1.09, 95% C.I of 1.02 – 1.16] respectively).

**Table 3:** Multivariate analysis of factors associated with non-adherence

**Variable**	**β**	**S.E.**	**Wald**	**p-value**	**Odds ratio with 95% C.I**
Marital status (single)	0.46	0.25	3.54	0.060	1.59(0.98-2.56)
High knowledge score	-0.14	0.06	6.27	0.012	0.87(0.78-0.97)
Presence of other co-morbid disease	0.42	0.31	1.83	0.177	1.53(0.83-2.81)
High number of medications consumed per day	0.08	0.07	1.33	0.249	1.08(0.95-1.24)
High Specific – necessity score	-0.07	0.03	5.77	0.016	0.93(0.88-0.99)
High Specific – concern score	0.07	0.03	11.86	0.001	1.10(1.04-1.16)
High General – harm score	0.07	0.03	7.29	0.007	1.09(1.02-1.16)

*Abbreviations*: *C.I *confidence interval, β coefficient of predictor variables, *S.E. *Standard error.

## Discussion

In the current study, medication adherence and its potential association with beliefs about medicines and diabetes–related knowledge was assessed in a selected sample of Arab Palestinians patients with type II DM. Our results showed that non-adherence was significantly associated with diabetes-related knowledge, beliefs about necessity of the anti-diabetic medications, concerns about adverse consequences of anti-diabetic medications, and beliefs that all medicines are essentially harmful. Our findings regarding association of medication adherence with knowledge and belief are in agreement with those obtained by other investigators. Most studies about medication adherence concluded that negative beliefs about medications is a powerful barrier to successful adherence [25-31]. Therefore, healthcare providers should address patient’s beliefs about medications in the hope of improving medication adherence and therapeutic outcome. Furthermore, healthcare workers need to assess and educate patients about diabetes mellitus to improve the level of medication adherence and consequently therapeutic outcome.

The results of our study showed that most diabetic patients strongly believe that anti-diabetic medications are necessary for their current and future health. However, diabetic patients expressed their concerns about the adverse consequences of taking anti-diabetic medications on regular basis. Similar findings were also reported in hypertensive patients [32]. Concerns of about long-term effects of taking anti-diabetic medications should be addressed by healthcare workers to minimize concerns and consequently improve medication adherence. Patients with diabetes mellitus need to know that their medications are not addictive and that medications have an acceptable safety profile for long-term use. Studies have reported that concerns about adverse consequences medications is independent of patients’ age and level of education [33]. Therefore, concerns about anti-diabetic medications need to be addressed regardless of the age or level of education of the patient. It is important to note that not all studies endorsed the finding that belief about medications is an important factor in medication adherence. For example, a Swedish study concluded that about one-third of the migraineurs did not adhere to their prophylactic drugs and that belief about medicines and medication-related factors could not predict non-adherence among those patients. The authors recommended further research on medication-related variables in relation to adherence among migraineurs [34]. These findings might suggest that the influence of beliefs on medication adherence is dependent on the type of the chronic disease.

An interesting finding in our study is the effect of total number of medications consumed daily on medication adherence among diabetic patients. Univariate analysis showed weak significant association between total number of medications and medication adherence. However, this weak significant association disappeared when strong factors like beliefs and disease-related knowledge were entered into the regression model. Furthermore, anti-diabetic regimen (mono versus combination therapy) showed no significant association with medication adherence in univariate analysis. This emphasizes the idea that it is not the total number of medications that determines level of adherence, rather, it is the belief about the importance and necessity of medicines for patient’s life which determines level of adherence. Similar result was obtained with regard to marital status. Univariate analysis showed that married patients were less likely to be non-adherent compared to single patients. However this association disappeared in multiple regression model. This means that family factors are important for medication adherence when considered as single factor but such family factors become insignificant when considered in the presence of other stronger factors like beliefs and disease-related knowledge.

In our study we used self-reporting method to measure medication adherence because it is considered the simplest and the least expensive method. George et al. found that when a valid scale [35], like Morisky questionnaire, is used to assess medication adherence, the obtained scores will be accurate with both sensitivity and specificity of over 70%. The 8-item Morisky Medication Adherence Questionnaire (MMAS-8^©^) has been translated into different languages and has been validated in patients with different types of chronic illnesses [23,36,37]. In our study, we used “Beliefs about Medicines Questionnaire (BMQ)” to measure patients’ beliefs about medicines. The BMQ has been translated into several languages to assess beliefs about medicines across a wide range of diseases like diabetes mellitus, mental health illnesses, rheumatoid arthritis and others [30], [33]. Therefore, tools used in our study are considered appropriate tools to achieve the stated objective.

Our study had few limitations. First, a self-report method was used to assess medication adherence. Therefore, overestimation of adherence may have occurred. More precise estimates of medication adherence can be obtained through direct methods. However, self-reported assessment of medication adherence is practical and inexpensive. Second, the validity of the Arabic version of adherence and belief scales has not been tested and further studies are needed to confirm validity of both scales among patients in the Arab world. Third, although the sample size was relatively large, it was not representative of Palestinian or Arab diabetic patients. Therefore, caution should be exercised in generalizing our findings. Fourth, the selection method might have created bias toward positive beliefs since patients who attend to the clinic are those who usually care about their health. These limitations might be responsible for the small observed odds ratio. Finally, no glycemic control data (HbA1C) were obtained. If such piece of information was available, then we would be able to link adherence, beliefs and knowledge with glycemic control.

## Conclusions

As a conclusion, beliefs in one’s medications and diabetes-related knowledge were significantly associated with adherence. Assessment of beliefs and knowledge can be used to understand variations in adherence among diabetic patients. The Beliefs about Medicines Questionnaire may be an appropriate tool to assess beliefs about medicines. Finally, improving knowledge of diabetic patients about their illness can positively influence their medication adherence and therapeutic outcome.

## Abbreviations

DM: Diabetes mellitus; MMAS-8©: Morisky Medication Adherence Scale; BMQ: Beliefs about medicines questionnaire; MDKT: Michigan diabetes knowledge test; IRB: Institutional review board; SPSS: Statistical Package for Social Sciences; Q1-Q3: Lower – Upper quartiles; SD: Standard deviation; O.R: Odds ratio; C.I: Confidence interval.

## Competing interests

The authors declare that they have no competing interests.

## Authors’ contributions

All authors were involved in drafting the article and all authors approved the final version to be submitted for publication. All authors have added an intellectual significant value to the manuscript. RA, MD, ME and SN were involved in subject recruitment and interview, data collection, data coding and entry, literature review, and manuscript editing. This was done as a Pharm D graduation project at An-Najah National University. WS, SA and SZ were involved in concept, study design, data analysis, data interpretation, manuscript writing and editing, manuscript submission, manuscript revision. The project was initially and originally conceptualized and designed by the Clinical Pharmacology/ Toxicology Research Group at An-Najah National University (WS, SZ, and SA). The project was then assigned to Pharm. D senior students as a graduation project and was academically supervised by W.S and S.Z in adherence to An-Najah University regulations with regard to academic supervision for graduation projects.

## Authors’ information

Professor Waleed M. Sweileh is the head of a research group (S.Z, S.A, A.S and A.A) which has published in the field of clinical pharmacology, toxicology, pharmacoepidemiology, social and community pharmacy, clinical pharmacy and medicine. The research group has also supervised many students in the fields of nursing, public health and pharmacy.

## Pre-publication history

The pre-publication history for this paper can be accessed here:

http://www.biomedcentral.com/1471-2458/14/94/prepub

## References

[B1] Shaw JE Sicree RA Zimmet PZ Global estimates of the prevalence of diabetes for 2010 and 2030 Diabetes Res Clin Pract 2009 87 1 4 14 19896746 10.1016/j.diabres.2009.10.007

[B2] Husseini A Abu-Rmeileh NM Mikki N Ramahi TM Ghosh HA Barghuthi N Khalili M Bjertness E Holmboe-Ottesen G Jervell J Cardiovascular diseases, diabetes mellitus, and cancer in the occupied Palestinian territory Lancet 2009 373 9668 1041 1049 10.1016/S0140-6736(09)60109-4 19268350

[B3] Sokol MC McGuigan KA Verbrugge RR Epstein RS Impact of medication adherence on hospitalization risk and healthcare cost Med Care 2005 43 6 521 530 10.1097/01.mlr.0000163641.86870.af 15908846

[B4] Ho PM Rumsfeld JS Masoudi FA McClure DL Plomondon ME Steiner JF Magid DJ Effect of medication nonadherence on hospitalization and mortality among patients with diabetes mellitus Arch Intern Med 2006 166 17 1836 1841 10.1001/archinte.166.17.1836 17000939

[B5] Vermeire E Hearnshaw H Van Royen P Denekens J Patient adherence to treatment: three decades of research. A comprehensive review J Clin Pharm Ther 2001 26 5 331 342 10.1046/j.1365-2710.2001.00363.x 11679023

[B6] Horne R Weinman J Patients’beliefs about prescribed medicines and their role in adherence to treatment in chronic physical illness J Psychosom Res 1999 47 6 555 567 10.1016/S0022-3999(99)00057-4 10661603

[B7] Horne R Weinman J Self-regulation and self-management in asthma: exploring the role of illness perceptions and treatment beliefs in explaining non-adherence to preventer medication Psychol Health 2002 17 1 17 32 10.1080/08870440290001502

[B8] Al-Qazaz H Sulaiman SA Hassali MA Shafie AA Sundram S Al-Nuri R Saleem F Diabetes knowledge, medication adherence and glycemic control among patients with type 2 diabetes Int J Clin Pharm 2011 33 6 1028 35 *Epub 2011 Nov 15* 2011 10.1007/s11096-011-9582-2 22083724

[B9] Jamous RM Sweileh WM Abu-Taha AS Sawalha AF Zyoud SH Morisky DE Adherence and satisfaction with oral hypoglycemic medications: a pilot study in Palestine Int J Clin Pharm 2011 33 6 942 948 10.1007/s11096-011-9561-7 21918840

[B10] Sweileh WM Ihbesheh MS Jarar IS Taha AS Sawalha AF Zyoud SH Jamous RM Morisky DE Self-reported medication adherence and treatment satisfaction in patients with epilepsy Epilepsy Behav 2011 21 3 301 305 10.1016/j.yebeh.2011.04.011 21576040

[B11] Horne R Patients’beliefs about treatment: the hidden determinant of treatment outcome? J Psychosom Res 1999 47 6 491 495 10.1016/S0022-3999(99)00058-6 10661596

[B12] Horne R Clatworthy J Polmear A Weinman J Do hypertensive patients’beliefs about their illness and treatment influence medication adherence and quality of life? J Hum Hypertens 2001 15 Suppl 1 S65 68 11685914 10.1038/sj.jhh.1001081

[B13] Al-Saeedi M Elzubier AG Bahnassi AA Al-Dawood KM Patterns of belief and use of traditional remedies by diabetic patients in Mecca, Saudi Arabia East Mediterr Health J 2003 9 1–2 99 107 15562738

[B14] Mann DM Ponieman D Leventhal H Halm EA Predictors of adherence to diabetes medications: the role of disease and medication beliefs J Behav Med 2009 32 278 284 10.1007/s10865-009-9202-y 19184390

[B15] Al-Qazaz HK Hassali MA Shafie AA Sulaiman SA Sundram S Morisky DE The eight-item Morisky Medication Adherence Scale MMAS: translation and validation of the Malaysian version Diabetes Res Clin Pract 2010 90 2 216 221 10.1016/j.diabres.2010.08.012 20832888 PMC3109726

[B16] Al-Qazaz HK Hassali MA Shafie AA Syed Sulaiman SA Sundram S Perception and knowledge of patients with type 2 diabetes in Malaysia about their disease and medication: A qualitative study Res Soc Adm Pharm 2011 7 2 180 191 10.1016/j.sapharm.2010.04.005 21272545

[B17] Fawzi W Abdel Mohsen MY Hashem AH Moussa S Coker E Wilson KC Beliefs about medications predict adherence to antidepressants in older adults Int Psychogeriatr 2012 24 1 159 169 10.1017/S1041610211001049 21729414

[B18] Zyoud SH Al-Jabi SW Sweileh WM Morisky DE Relationship of treatment satisfaction to medication adherence: findings from a cross-sectional survey among hypertensive patients in Palestine Health Qual Life Outcomes 2013 11 1 191 10.1186/1477-7525-11-191 24195638 PMC4228307

[B19] Zyoud SH Al-Jabi SW Sweileh WM Wildali AH Saleem HM Aysa HA Badwan MA Awang R Morisky DE Health-related quality of life associated with treatment adherence in patients with hypertension: a cross-sectional study Int J Cardiol 2013 168 3 2981 2983 10.1016/j.ijcard.2013.04.105 23647601

[B20] Sweileh WM Ihbesheh MS Jarar IS Sawalha AF Abu Taha AS Zyoud SH Morisky DE Differences in medication adherence, satisfaction and clinical symptoms in schizophrenic outpatients taking different antipsychotic regimens Curr Drug Saf 2011 6 5 285 290 10.2174/157488611798918755 22424535

[B21] Sweileh WM Ihbesheh MS Jarar IS Sawalha AF Abu Taha AS Zyoud SH Morisky DE Antipsychotic medication adherence and satisfaction among Palestinian people with schizophrenia Curr Clin Pharmacol 2012 7 1 49 55 10.2174/157488412799218761 22299769

[B22] Daniel WW Biostatistics: Basic Concepts and Methodology for the Health Sciences 2010 New Jersey, USA: John Wiley & Sons

[B23] Morisky DE Green LW Levine DM Concurrent and predictive validity of a self-reported measure of medication adherence Med Care 1986 24 1 67 74 10.1097/00005650-198601000-00007 3945130

[B24] Fitzgerald JT Funnell MM Hess GE Barr PA Anderson RM Hiss RG Davis WK The reliability and validity of a brief diabetes knowledge test Diabetes Care 1998 21 5 706 710 10.2337/diacare.21.5.706 9589228

[B25] Gatti ME Jacobson KL Gazmararian JA Schmotzer B Kripalani S Relationships between beliefs about medications and adherence Am J Health Syst Pharm 2009 66 7 657 664 10.2146/ajhp080064 19299373

[B26] Chummun H Boland D How patient beliefs affect adherence to prescribed medication regimens Br J Nurs 2013 22 5 270 276 23545553 10.12968/bjon.2013.22.5.270

[B27] Clifford S Barber N Horne R Understanding different beliefs held by adherers, unintentional nonadherers, and intentional nonadherers: application of the Necessity-Concerns Framework J Psychosom Res 2008 64 1 41 46 10.1016/j.jpsychores.2007.05.004 18157998

[B28] Iihara N Tsukamoto T Morita S Miyoshi C Takabatake K Kurosaki Y Beliefs of chronically ill Japanese patients that lead to intentional non-adherence to medication J Clin Pharm Ther 2004 29 5 417 424 10.1111/j.1365-2710.2004.00580.x 15482384

[B29] Mardby AC Akerlind I Jorgensen T Beliefs about medicines and self-reported adherence among pharmacy clients Patient Educ Couns 2007 69 1–3 158 164 17913439 10.1016/j.pec.2007.08.011

[B30] Menckeberg TT Bouvy ML Bracke M Kaptein AA Leufkens HG Raaijmakers JA Horne R Beliefs about medicines predict refill adherence to inhaled corticosteroids J Psychosom Res 2008 64 1 47 54 10.1016/j.jpsychores.2007.07.016 18157999

[B31] Sirey JA Greenfield A Weinberger MI Bruce ML Medication beliefs and self-reported adherence among community-dwelling older adults Clin Ther 2013 35 2 153 160 10.1016/j.clinthera.2013.01.001 23357585 PMC3603227

[B32] Ruppar TM Dobbels F De Geest S Medication beliefs and antihypertensive adherence among older adults: a pilot study Geriatr Nurs 2012 33 2 89 95 10.1016/j.gerinurse.2012.01.006 22387190

[B33] Neame R Hammond A Beliefs about medications: a questionnaire survey of people with rheumatoid arthritis Rheumatology (Oxford) 2005 44 6 762 767 10.1093/rheumatology/keh587 15741193

[B34] Hedenrud T Jonsson P Linde M Beliefs about medicines and adherence among Swedish migraineurs Ann Pharmacother 2008 42 1 39 45 18073328 10.1345/aph.1K354

[B35] George CF Peveler RC Heiliger S Thompson C Compliance with tricyclic antidepressants: the value of four different methods of assessment Br J Clin Pharmacol 2000 50 2 166 171 10.1046/j.1365-2125.2000.00244.x 10930969 PMC2014396

[B36] Korb-Savoldelli V Gillaizeau F Pouchot J Lenain E Postel-Vinay N Plouin PF Durieux P Sabatier B Validation of a French version of the 8-item Morisky medication adherence scale in hypertensive adults J Clin Hypertens (Greenwich) 2012 14 7 429 434 10.1111/j.1751-7176.2012.00634.x 22747615 PMC8108765

[B37] Sakthong P Chabunthom R Charoenvisuthiwongs R Psychometric properties of the thai version of the 8-item morisky medication adherence scale in patients with type 2 diabetes Ann Pharmacother 2009 43 5 950 957 10.1345/aph.1L453 19366872

